# Machine Learning-based Prediction of Active Tuberculosis in People With HIV Using Clinical Data

**DOI:** 10.1093/cid/ciaf149

**Published:** 2025-03-25

**Authors:** Lena Bartl, Marius Zeeb, Marisa Kälin, Tom Loosli, Julia Notter, Hansjakob Furrer, Matthias Hoffmann, Hans H Hirsch, Robert Zangerle, Katharina Grabmeier-Pfistershammer, Michael Knappik, Alexandra Calmy, Jose Damas Fernandez, Niklaus D Labhardt, Enos Bernasconi, Huldrych F Günthard, Roger D Kouyos, Katharina Kusejko, Johannes Nemeth

**Affiliations:** Department of Infectious Diseases and Hospital Epidemiology, University Hospital Zurich, Zurich, Switzerland; Department of Infectious Diseases and Hospital Epidemiology, University Hospital Zurich, Zurich, Switzerland; Institute of Medical Virology, University of Zurich, Zurich, Switzerland; Department of Infectious Diseases and Hospital Epidemiology, University Hospital Zurich, Zurich, Switzerland; Department of Infectious Diseases and Hospital Epidemiology, University Hospital Zurich, Zurich, Switzerland; Institute of Medical Virology, University of Zurich, Zurich, Switzerland; Division of Infectious Diseases, Infection Prevention and Travel Medicine, Cantonal Hospital St. Gallen, St. Gallen, Switzerland; Department of Infectious Diseases, Inselspital, Bern University Hospital, University of Bern, Bern, Switzerland; Division of Infectious Diseases and Hospital Epidemiology, Cantonal Hospital Olten, Olten, Switzerland; Transplantation & Clinical Virology, University of Basel, Basel, Switzerland; Department of Dermatology, Venereology and Allergy, Medical University Innsbruck, Innsbruck, Austria; Department of Dermatology, Medical University Vienna, Vienna, Austria; Department of Respiratory Medicine, Klinik Penzing, Vienna, Austria; HIV Unit, Division of Infectious Diseases, University Hospital Geneva, University of Geneva, Geneva, Switzerland; Division of Infectious Diseases, University Hospital Lausanne, University of Lausanne, Lausanne, Switzerland; Division Clinical Epidemiology, Department of Clinical Research, University Hospital Basel, Basel, Switzerland; Division of Infectious Diseases, Ente Ospedaliero Cantonale, Lugano, Switzerland; Department of Infectious Diseases and Hospital Epidemiology, University Hospital Zurich, Zurich, Switzerland; Institute of Medical Virology, University of Zurich, Zurich, Switzerland; Department of Infectious Diseases and Hospital Epidemiology, University Hospital Zurich, Zurich, Switzerland; Institute of Medical Virology, University of Zurich, Zurich, Switzerland; Department of Infectious Diseases and Hospital Epidemiology, University Hospital Zurich, Zurich, Switzerland; Institute of Medical Virology, University of Zurich, Zurich, Switzerland; Department of Infectious Diseases and Hospital Epidemiology, University Hospital Zurich, Zurich, Switzerland

**Keywords:** tuberculosis, HIV, clinical risk score, machine learning, prediction

## Abstract

**Background:**

Coinfections of *Mycobacterium tuberculosis* (MTB) and human immunodeficiency virus (HIV) impose a substantial global health burden. Patients with MTB infection face a heightened risk of progression to incident active TB, which preventive therapy can mitigate. Current testing methods often fail to identify individuals who subsequently develop incident active TB.

**Methods:**

We developed random forest models to predict incident active TB using patients' medical data at HIV-1 diagnosis. Training our model involved using clinical data routinely collected at enrollment from the Swiss HIV Cohort Study (SHCS). This dataset encompassed 55 people with HIV (PWH) who developed incident active TB 6 months after enrollment and 1432 matched PWH without TB enrolled between 2000 and 2023. External validation used data from the Austrian HIV Cohort Study, comprising 43 people with incident active TB and 1005 people without TB.

**Results:**

We predicted incident active TB with an area under the receiver operating characteristic curve of 0.83 (95% CI: .8–.86) in the SHCS. After adjusting for ethnicity and the region of origin and refitting the model with fewer parameters, we obtained comparable receiver operating characteristic curve values of 0.72 (SHCS) and 0.67 (Austrian HIV Cohort Study). Our model outperformed the standard of care (tuberculin skin test and interferon-gamma release assay) in identifying high-risk patients, demonstrated by a lower number needed to diagnose (1.96 vs 4).

**Conclusions:**

Models based on machine learning offer considerable promise for improving care for PWH, requiring no additional data collection and incurring minimal additional costs while enhancing the identification of PWH that could benefit from preventive TB treatment.

## Teaser

Machine learning models can predict incident active tuberculosis in people with HIV (PWH) using routine medical data, outperforming current tests. This may improve clinical management of PWH with minimal added cost and no extra data collection.

Human immunodeficiency virus 1 (HIV-1) and *Mycobacterium tuberculosis* (MTB) infection synergistically cause mortality, with HIV-1 significantly influencing the progression of MTB infection to incident active TB [1]. Although the introduction of antiretroviral therapy (ART) has dramatically reduced the risk of HIV-1–associated active TB, people with HIV-1 (PWH) are still at increased risk of developing active TB. This increase is in particular pronounced in the case of late diagnosis of the HIV-1 infection or restricted access to ART due to factors such as socioeconomic disruption, war, and refugee status [2–4]. Current diagnostic TB tests rely on T-cell responses, such as interferon-gamma release assay (IGRA) and tuberculin skin test (TST). However, in PWH, T cells exhibit impaired functionality, both quantitatively and qualitatively, which makes it challenging to predict TB in these high-risk populations [5, 6].

This study aimed to devise a prognostic score for predicting the occurrence of “incident active tuberculosis (TB)”, defined as active TB that arises at least 6 months after HIV-1 diagnosis. Our model is built on clinical data routinely collected during the initial consultation at HIV-1 diagnosis, with the 6-month window chosen to enable effective preventive treatment to mitigate the onset of active TB [7].

Artificial intelligence (AI) approaches specifically machine learning, have emerged as promising tools in healthcare for detecting novel patterns in already available data [8, 9]. In the context of predicting TB in PWH, machine learning techniques offer the potential to identify indicators and risk factors that may not be identified through traditional statistical methods. A few examples include the approach of identifying incident active TB using clinical parameters [10], early prediction of TB transmission [11] as well as treatment outcome [12]. The field of machine learning was also applied to many other fields in TB and types of data, providing new perspectives and possibilities in healthcare [13–15].

However, the successful implementation of AI approaches depends on the availability of high-quality input data [9, 16]. The Swiss HIV Cohort Study (SHCS) meticulously records longitudinal clinical and laboratory parameters of PWH in Switzerland, using an elaborate high-quality workflow for semiautomated data entry during the regular biannual follow-ups. This generates a research database consisting of highly accurate and consistent data. Furthermore, as more than 70% of newly diagnosed PWH are enrolled in the study, this results in a large and representative study population [17]. Similarly, the Austrian HIV Cohort Study (AHIVCOS) serves as a valuable external validation cohort [18], offering a dataset structured similarly to the SHCS. In this study, 2 datasets were used to develop an AI-based diagnostic tool for predicting TB in PWH. This novel approach enables the identification of individuals at risk of progressing to active TB within a high-risk population, operating independently of the current gold-standard (IGRA/TST).

## METHODS

### Participants

The study population consists of PWH enrolled in the SHCS, a multicentric Swiss study launched in 1988 with a cumulative number of more than 21 000 participants. The SHCS collects longitudinal information on demographic, clinical, behavioral, and laboratory variables in at least biannual follow-up visits. External validation involved individuals from the AHIVCOS, a multicentric cohort of PWH in Austria [17, 18].

### Definitions

The diagnosis of active TB relied on clinical signs, symptoms (eg, coughing), indicative X-rays or other imaging, and subsequent microbiological detection of MTB [17, 19]. Active TB diagnoses were categorized as prevalent TB (identified at SHCS enrollment or <6 months after enrollment) and incident active TB (TB diagnosis >6 months after enrollment). Latent MTB infection was defined as a positive TST or IGRA test at least 6 months before the occurrence of incident TB. Sensitivity and specificity of MTB testing was estimated based on SHCS data from 2000 through 2023 [5]. Preventive treatment entailed initiating rifampicin, rifabutin, isoniazid, rifapentine, or pyrazinamide therapy at any point during the SHCS follow-up [5].

### Selection of the Population and Variables

Since the SHCS started collecting in-depth demographic and clinical variables in 2000, all participants registered in the SHCS between 1 January 2000 and 1 November 2023 were included in this study. All people with prevalent TB (ie, TB diagnosed at cohort enrollment) were excluded. Participants preventively treated for MTB infection were also excluded. People with incident active TB were defined as patients with incident TB occurring at least 6 months after enrollment. A comprehensive range of demographic, laboratory, and lifestyle variables was included; variables with missing data for more than 35% of the study population were excluded. All included variables are listed in Supplementary Table 1, variables that did not meet the criteria and were thus excluded are listed in Supplementary Table 2. World Health Organization regions were used to recode regions for simplicity [20]. The data used in the model were obtained during the initial SHCS enrollment consultations, with additional data from clinical consultations conducted 6 months before or after enrollment. The model did not include MTB testing. Any participants with more than 35% missing data for the chosen clinical variables were excluded from our analysis. Matching of people without TB was solely based on the registration year to account for advancements in HIV patient care, and to enhance model robustness, 30 people without TB were selected per person with incident active TB.

### Outcome

The primary outcome was predicting incident TB development. The binary classification was assessed using the area under the receiver operating characteristics (ROC) curve (AUC) and Youden Index. Secondary outcomes included variable importance analysis, the impact on the number needed to diagnose to have a comparison with the current standard of testing, as well as the area under the precision-recall curve (AUPRC) to assess the predictive power for incident active TB, which can give more insight on model precision when using unbalanced data sets [21].

### Model Creation

We constructed a random forest algorithm using the *randomForest* package in R [22–24] (see Supplementary Material for details). The data were partitioned into training (70%) and validation (30%) sets using the *caret* package in R [25], where the validation set was kept out of the model building to be a valid control dataset to assess model performance. The split was stratified by people with incident active TB and people without TB to ensure a balanced class distribution within the splits based on the outcome. Imputation of missing data was performed separately on the training and validation datasets using again the *randomForest* package in R. Imputation was conducted iteratively with random seed selection, using 10 iterations. The random forest was trained using the training dataset with internal cross-validation with 5 splits and 100 iterations, each iteration creating a new training and testing dataset, to achieve optimum model performance. Model performance was assessed using a validation set previously unknown to the model. We repeated imputation and model creation 1000 times, each with a distinct randomly chosen seed. This procedure aimed to provide a more accurate estimate of performance by pooling results from different forests and calculating the mean ROC of the AUC and variable importance [26]. Optimal model performance resulted from using seven features in each tree and 500 trees for each forest.

### Statistical Analysis

Performance assessment involved using the *pROC* package [27] in R to generate ROC curves for each forest, followed by combination and calculation of mean curves. The ROC curve was smoothed using the *geomsmooth*-function of *ggplot2* [28]. Optimal points were determined using the Youden Index (sensitivity + specificity − 1) for comparison with the clinical standard (both TST and IGRA during 2000–2023). This was then expressed in a number needed to diagnose (NND), which signifies the number of people with a specific illness that must undergo screening by the test to yield 1 correct diagnosis (ie, true positive and positive predictive value [PPV]). Reported AUC results were obtained using the test set. Furthermore, we used the *PRROC* package in R to generate the AUPRC curves [29].

### External Validation

The model underwent external validation by training it on SHCS data and validating it with both SHCS and AHIVCOS data in separate analyses. Data preparation steps for the AHIVCOS mirrored those employed for SHCS data. We refitted the model using solely the most important variables, previously identified as the top 20 predictors based on variable importance that were available in the AHIVCOS database. Additionally, we excluded the variables “Ethnicity” and “Region of Origin” for the external validation only because these parameters were distributed differently between the cohorts. We instead looked at the distribution of low- and high-incidence TB countries among the participants. Model performance was assessed using the AUC of the ROC curve and variable importance analysis.

## RESULTS

### Study Population

**Figure 1.**
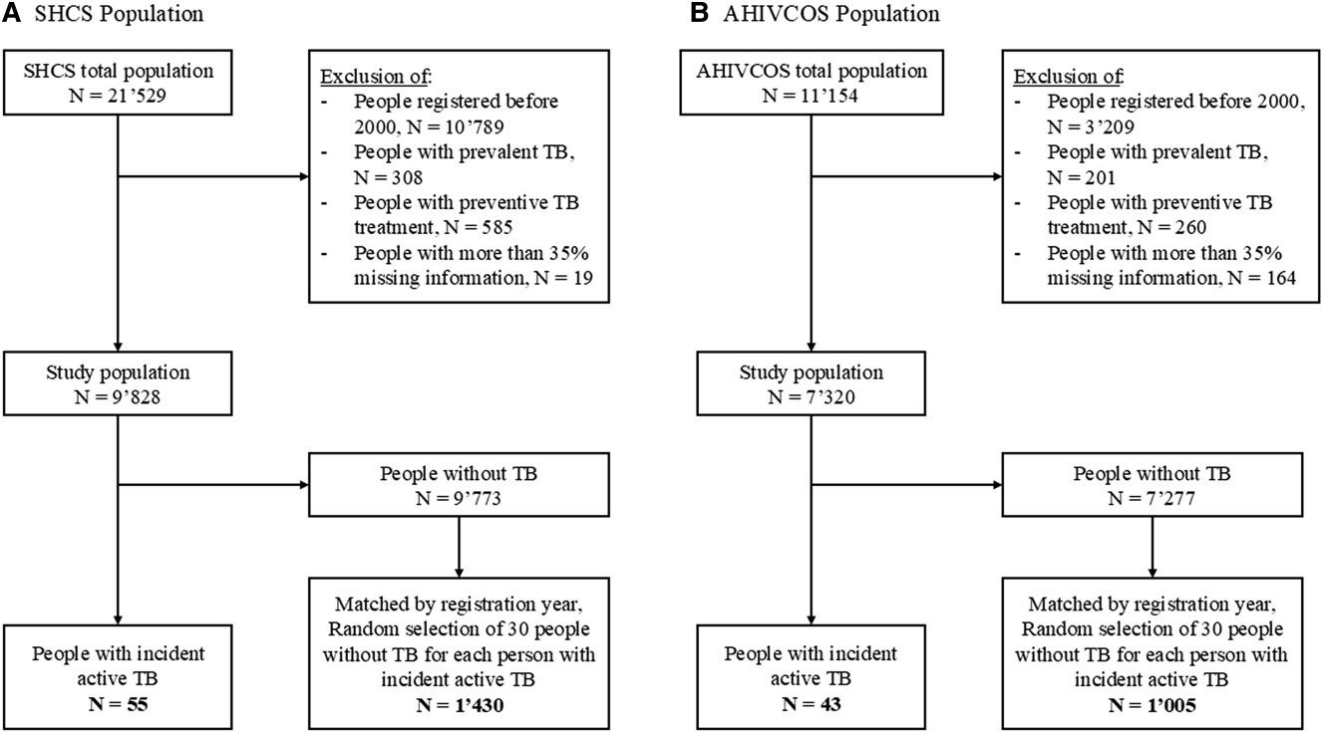
Selection of the population for the SHCS and the AHIVCOS. Flowchart of the study populations: *A*, SHCS and *B*, AHIVCOS. Incident active TB, TB outbreak ≥6 m after registration. Abbreviations: AHIVCOS, Austrian HIV Cohort Study; HIV, human immunodeficiency virus; SHCS, Swiss HIV Cohort Study; TB, tuberculosis.

Among the 21 529 individuals enrolled in the SHCS, those registered before the year 2000, people with prevalent TB, or individuals treated preventively for MTB infection were excluded. This led to the inclusion of 9828 individuals in our study population, with 55 individuals identified as people with incident active TB, 9773 identified as people without TB. After matching (see Methods), the study population comprised 1430 individuals, consisting of 1029 (72.0%) males and 401 (28.0%) females (Figure 1). The majority (1138, 79.6%) were White, with a mean age of 38.2 years.

For external validation of the derived score, AHIVCOS data were used to determine the reproducibility and predictive power of our model in a different study setting [30]. Of the 11 154 patients included in AHIVCOS, 43 people with incident active TB and 1005 people without TB were included in the validation dataset (Figure 1, Table 1). Overall, data from 2533 participants from both cohorts were used, with 98 people with incident active TB.

**Table 1.** Characteristics of PWH Included in the Random Forest Model Analysis During 6 Months Before or After Their Registration in the SHCS and AHIVCOS

	Total		SHCS		AHIVCOS	
	People Without TB (N = 2435)	People With Incident Active TB (N = 98)	People Without TB (N = 1430)	People With Incident Active TB (N = 55)	People Without TB (N = 1005)	People With Incident Active TB (N = 43)
Sex						
Male	1774 (72.9%)	49 (50.0%)	1029 (72.0%)	24 (43.6%)	745 (74.1%)	25 (58.1%)
Female	660 (27.1%)	49 (50.0%)	401 (28.0%)	31 (56.4%)	259 (25.8%)	18 (41.9%)
Missing	1 (0.0%)	0 (0%)	0 (0%)	0 (0%)	1 (0.1%)	0 (0%)
Age (y)						
Mean [SD]	37.6 [10.7]	34.2 [10.3]	38.2 [9.99]	34.3 [9.46]	36.9 [11.5]	34.1 [11.4]
BMI (kg/m^2^)						
Mean [SD]	23.4 [3.87]	22.5 [3.63]	23.5 [3.67]	22.1 [3.62]	23.3 [4.24]	23.1 [3.63]
Missing	310 (12.7%)	18 (18.4%)	30 (2.1%)	3 (5.5%)	280 (27.9%)	15 (34.9%)
Region						
Americas	103 (4.2%)	3 (3.1%)	78 (5.5%)	3 (5.5%)	25 (2.5%)	0 (0%)
Western Pacific	18 (0.7%)	1 (1.0%)	17 (1.2%)	1 (1.8%)	1 (0.1%)	0 (0%)
Africa	276 (11.3%)	42 (42.9%)	155 (10.8%)	27 (49.1%)	121 (12.0%)	15 (34.9%)
Eastern Mediterranean	24 (1.0%)	1 (1.0%)	5 (0.3%)	0 (0%)	19 (1.9%)	1 (2.3%)
Southeast Asia	71 (2.9%)	9 (9.2%)	40 (2.8%)	6 (10.9%)	31 (3.1%)	3 (7.0%)
Europe	1940 (79.7%)	42 (42.9%)	1135 (79.4%)	18 (32.7%)	805 (80.1%)	24 (55.8%)
Missing	3 (0.1%)	0 (0%)	0 (0%)	0 (0%)	3 (0.3%)	0 (0%)
Ethnicity						
White	1901 (78.1%)	39 (39.8%)	1138 (79.6%)	16 (29.1%)	763 (75.9%)	23 (53.5%)
Black	293 (12.0%)	43 (43.9%)	184 (12.9%)	30 (54.5%)	109 (10.8%)	13 (30.2%)
Hispanic	63 (2.6%)	2 (2.0%)	55 (3.8%)	2 (3.6%)	8 (0.8%)	0 (0%)
Asian	81 (3.3%)	10 (10.2%)	53 (3.7%)	7 (12.7%)	28 (2.8%)	3 (7.0%)
Other	16 (0.7%)	0 (0%)	0 (0%)	0 (0%)	16 (1.6%)	0 (0%)
Unknown	1 (0.0%)	0 (0%)	0 (0%)	0 (0%)	1 (0.1%)	0 (0%)
Missing	80 (3.3%)	4 (4.1%)	0 (0%)	0 (0%)	80 (8.0%)	4 (9.3%)
CD4 count (cells/µL)						
Mean [SD]	409 [276]	367 [233]	410 [265]	354 [227]	408 [292]	385 [243]
Missing	4 (0.2%)	1 (1.0%)	0 (0%)	0 (0%)	4 (0.4%)	1 (2.3%)
Leukocytes (cells/µL)						
Mean [SD]	5720 [2510]	5260 [2020]	5510 [2340]	4960 [1850]	6020 [2720]	5680 [2200]
Missing	51 (2.1%)	4 (4.1%)	2 (0.1%)	0 (0%)	49 (4.9%)	4 (9.3%)
Cholesterol (mmol/L)						
Mean [SD]	4.62 [1.23]	4.33 [1.16]	4.69 [1.23]	4.27 [0.987]	4.52 [1.22]	4.41 [1.35]
Missing	142 (5.8%)	8 (8.2%)	85 (5.9%)	5 (9.1%)	57 (5.7%)	3 (7.0%)
Creatinine (µmol/L)						
Mean [SD]	77.6 [19.6]	69.7 [20.4]	78.0 [17.8]	68.5 [18.2]	77.0 [21.4]	70.8 [22.6]
Missing	372 (15.3%)	16 (16.3%)	351 (24.5%)	14 (25.5%)	21 (2.1%)	2 (4.7%)
RNA (log 10 copies)						
Mean [SD]	3.71 [1.86]	3.86 [1.53]	3.32 [1.98]	3.63 [1.55]	4.25 [1.52]	4.16 [1.47]
Missing	9 (0.4%)	0 (0%)	2 (0.1%)	0 (0%)	7 (0.7%)	0 (0%)
Hemoglobin (g/dL)						
Mean [SD]	13.7 [1.88]	12.6 [1.88]	13.7 [1.83]	12.4 [1.90]	13.7 [1.96]	12.9 [1.84]
Missing	43 (1.8%)	3 (3.1%)	3 (0.2%)	0 (0%)	40 (4.0%)	3 (7.0%)
Tuberculin skin test						
Positive	24 (1.0%)	5 (5.1%)	22 (1.5%)	5 (9.1%)	2 (0.2%)	0 (0%)
Negative	585 (24.0%)	16 (16.3%)	542 (37.9%)	15 (27.3%)	43 (4.3%)	1 (2.3%)
Uncertain	3 (0.1%)	0 (0%)	3 (0.2%)	0 (0%)	0 (0%)	0 (0%)
Missing	1823 (74.9%)	77 (78.6%)	863 (60.3%)	35 (63.6%)	960 (95.5%)	42 (97.7%)
Interferon-Gamma test						
Positive	15 (0.6%)	10 (10.2%)	1 (0.1%)	6 (10.9%)	14 (1.4%)	4 (9.3%)
Negative	310 (12.7%)	7 (7.1%)	118 (8.3%)	1 (1.8%)	192 (19.1%)	6 (14.0%)
Uncertain	13 (0.5%)	0 (0%)	3 (0.2%)	0 (0%)	10 (1.0%)	0 (0%)
Missing	2097 (86.1%)	81 (82.7%)	1308 (91.5%)	48 (87.3%)	789 (78.5%)	33 (76.7%)

Abbreviations: AHIVCOS, Austrian HIV Cohort Study; BMI, body mass index; SD, standard deviation; SHCS, Swiss HIV Cohort Study; PWH, people with HIV; TB, tuberculosis.

### Primary Outcome

**Figure 2.**
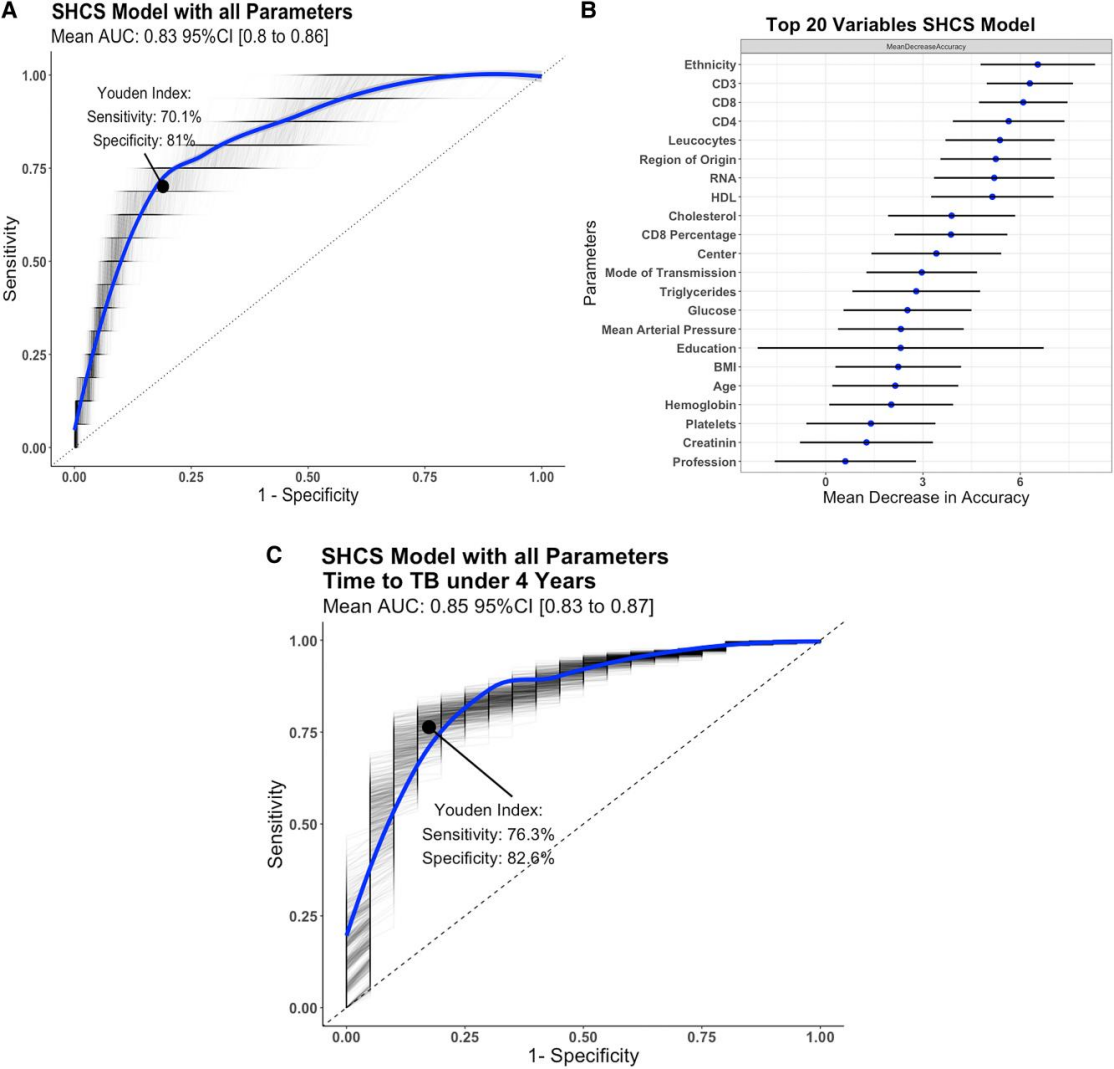
*A*, Receiver operating characteristic (ROC) curve for incident active TB outbreak derived from the random forest model prediction and true TB status, built on all parameters of the SHCS data. The Youden Index is calculated by (sensitivity + specificity − 1) and was used to determine the optimum point of the ROC curve. *B*, Variable importance sorted by mean decrease accuracy, measured by removing the association between a predictor variable and the outcome variable and determining the resulting increase in error. *C*, ROC curve for the incident active TB outbreak of the random forest model. The model was built using all parameters of the SHCS data and validated with SHCS data from patients who had their incident active TB outbreak less than 4 y after registration. Abbreviations: BMI, body mass index; CD, cluster of differentiation; HDL, high-density lipoproteins; SHCS, Swiss HIV Cohort Study; TB, tuberculosis.

We developed a random forest model to predict incident active TB in PWH within the SHCS, with 48 predictors (Supplementary Table 1). The mean AUC for the ROC curve predicting incident active TB in the test dataset was 0.83 (95% confidence interval: .8–.86). The sensitivity was 70.1% and the specificity was 81.0%, with a Youden index of 0.51 (Figure 2*A*). The AUPRC, which looks at the fraction of predicted positives among true positives, was 0.168 at a baseline of 0.03 (Supplementary Figure 1).

We assessed variable importance by mean decrease in accuracy, quantified by removing the association between predictor and outcome variables, and evaluated the error increase (Figure 2*B*). Demographic parameters, particularly region of origin and ethnicity, significantly influenced model performance due to varying TB incidence rates. Removing information on ethnicity and region of origin even decreased the predictive power to an AUC of 0.63 (Supplementary Figure 2) and adding TB testing (IGRA and TST) did not improve the model (Supplementary Figure 3). Socioeconomic status, represented by profession and education, and mode of transmission also impacted predictions. Laboratory results, including HIV-related metrics such as CD4 cell count and RNA levels, reflecting immune system status, played a major role in TB outbreak timing. Overall health indicators (body mass index [BMI], CD4, hemoglobin, etc.) also affected model accuracy and showed a statistically significant association with predicting incident active TB **(**Supplementary Figure 4).

We selected the top 20 predictors to simplify model application to external datasets. This reduced set yielded an AUC of 0.74, making it feasible for use with SHCS data and external validation (Supplementary Figure 5).

### External Validation

**Figure 3.**
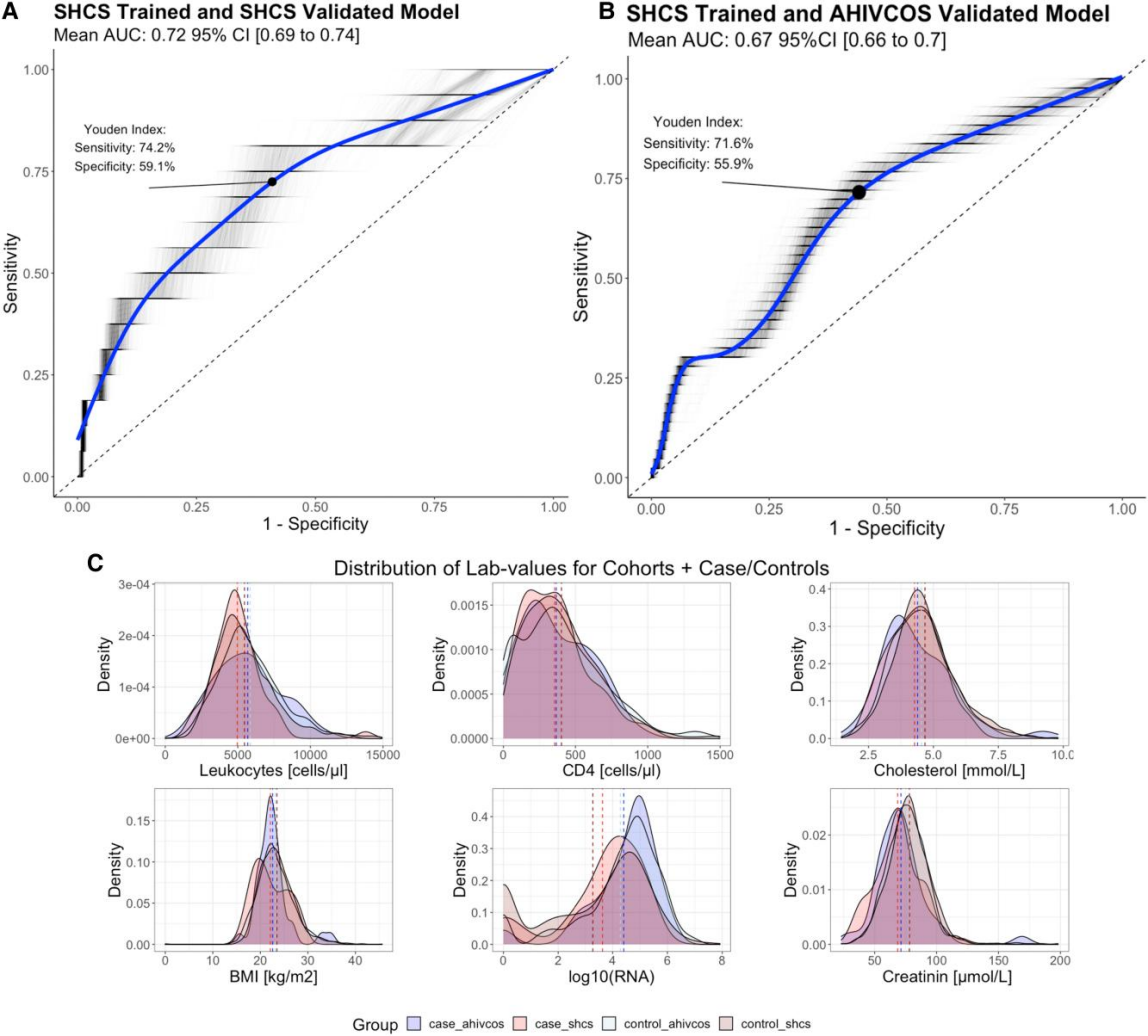
*A*, Receiver operating characteristic curve for incident active TB outbreak of the random forest model, built on the top 20 parameters excluding ethnicity and region of origin and recoding them into high- and low-incidence TB countries of the SHCS data, validated on SHCS data. The Youden Index was used to determine the optimum point of the ROC curve. *B*, Smoothed ROC curve for the model validated on AHIVCOS data. *C*, Variable distribution in the SHCS and AHIVCOS data, stratified by people with incident active TB and people without TB. For each group (SHCS people with v TB/people without TB, AHIVCOS people with incident active TB/people without TB), we examined the distribution of leukocytes, CD4 cell count, cholesterol, BMI, HIV RNA, and creatinine from left to right and top to bottom. The lines represent the mean of each group for each laboratory value. Abbreviations: AHIVCOS, Austrian HIV Cohort Study; AUC, area under the curve; BMI, body mass index; SHCS, Swiss HIV Cohort Study.

For the external validation, we have adapted the ethnicity variable included in the model, as the 2 cohorts differed considerably in this regard (Table 1). Northern America and both Western and Northern Europe were categorized as regions of “low incidence,” whereas the rest of the world was considered as “high incidence.” We thereby accounted for this disparity, which likely stems from differential immigration patterns, with people with incident active TB in Austria predominantly from Eastern Europe and in Switzerland predominantly from Africa. A comparable trend was observed for the region of origin (Table 1). Reapplying the model to SHCS data using the updated variables and validating on combined SHCS and AHIVCOS data resulted in AUCs for SHCS validation of 0.72, with a Youden Index of 0.33, sensitivity of 74.2%, and specificity of 59.1%. The AUC for AHIVCOS data was 0.67, with a Youden Index of 0.28, sensitivity of 71.6%, and specificity of 55.9% (Figures 3*A* and 3*B*). Of note, applying the model trained using the original ethnicity variable proved ineffective for the AHIVCOS with an AUC of 0.5 in the validation [18] (Supplementary Figure 6). For the externally validated score, we observed significant influences on predicting future incident active TB, of immune system parameters and variables indicative of patients' well-being at registration, such as BMI or creatinine levels (Supplementary Figure 7). For this analysis, we also looked at the AUPRC for SHCS data (0.111) and the AHIVCOS data (0.095) at a baseline of 0.03 (Supplementary Figure 8*A* and 8*B*).

### Time to TB Analysis

We examined the time from SHCS registration to incident active TB and its impact on the model's predictive power. We selected a 4-year cutoff as this was the average time for people with incident active TB to develop TB (Supplementary Figure 9*A* and 9*B*). The model performed better for shorter time to incident active TB. This likely occurs because biological changes, observable in laboratory and blood results, emerge closer to the outbreak.

### Comparison With the Current Gold Standard

In the SHCS from 2000–2023, both TST and IGRA were used for MTB infection testing. The sensitivity and specificity of MTB infection testing for predicting incident active TB were 30% and 94%, respectively [5]. MTB infection testing yielded a NND of 4, whereas our newly derived method based on the complete SHCS model required only 1.96 people with incident active TB to correctly identify 1, doubling the likelihood of diagnosing a person with incident active TB correctly. External validation also demonstrated a better result than MTB infection testing, with a NND of 3.6. PPV across all models was also better than MTB infection testing, with the complete model having a PPV of 10.3%, the external validation of 4.9%, and the testing standard of IGRA and TST of 2.7% and 1.5%, respectively [31] (Table 2).

**Table 2.** Model Used, Along With the Data Employed for Training and Validation, the Number Needed to Diagnose—Indicating How Many People With the Disease Must Be Screened by This Diagnostic Method to Identify 1 True Positive—and the AUC of Each Corresponding Model

Model	Training Data	Validation Data	Number Needed to Diagnose	Mean Area Under the Curve	Positive Predictive Value (PPV)
SHCS total	SHCS with all parameters	SHCS with all parameters	1.96	0.83	10.3%
SHCS external	SHCS top 20 with adjusted variable for region and ethnicity “incidence”	SHCS top 20 with adjusted variable for region and ethnicity “incidence”	2.99	0.72	5.3%
AHIVCOS external	SHCS top 20 with adjusted variable for region and ethnicity “incidence”	AHIVCOS top 20 with adjusted variable for region and ethnicity “incidence”	3.64	0.67	4.9%
SHCS time to TB younger than age 4 y	SHCS people with incident active TB with time to TB younger than age 4 y with all parameters	SHCS with all parameters	1.70	0.85	14.4%
MTB infection testing (IGRA and TST combined)	Clinical standard	Clinical standard	4.00	…	…

The table also includes the PPV at the prevalence used during model creation (0.03) and the sensitivity and specificity at the Youden Index point for each model.

Abbreviations: AHIVCOS, Austrian HIV Cohort Study; AUC, area under the curve; IGRA, interferon-gamma release assay; MTB infection, *Mycobacterium tuberculosis* infection; SHCS, Swiss HIV Cohort Study; TST, tuberculin skin test.

## DISCUSSION

Conventional testing methods often lack efficacy in identifying individuals who subsequently develop incident active TB, particularly among PWH [6, 32]. In response to this challenge, we developed a machine learning model that uses clinical data from PWH at HIV-1 diagnosis to predict incident active TB. The model performed at least as well as the clinical standard in predicting incident active TB in the SHCS. This performance was subsequently validated in the AHIVCOS, an independent external validation cohort, confirming the robustness of our model. Our approach offers a surprisingly simple but compelling example of how AI can be used for predicting relatively rare events, such as the onset of incident active TB in PWH.

Our machine learning approach functions independently of T-cell activity, effectively overcoming a key limitation of IGRA-based prediction systems that depend heavily on T-cell responses. This reliance is particularly problematic in PWH, where both reduced T-cell quantity and quality frequently result in false-negative outcomes due to impaired T-cell responsiveness [5, 33]. While previous attempts to predict incident active TB using clinical parameters (with and without IGRA) have been made, these efforts were limited in scale and duration compared to our project [34–36].

The inclusion of factors such as CD4 count and HIV-1 viral load in our diagnostic score, recognized risk factors for TB development, strengthens the validity of our findings [37]. Moreover, the identification of novel metabolism-associated factors is notable. For instance, the predictive value of low creatinine levels suggests compromised muscle mass and nutritional status. Similarly, the influence of high-density lipoprotein and triglycerides underscores metabolic perturbations in individuals at high risk of active incident TB. Poor nutrition has long been acknowledged as a TB risk factor [38], as evidenced by recent large-scale trials in India [39] and by the World Health Organization [40].

Using existing data offers cost savings, as IGRA tests are expensive, and TST requires multiple clinical visits. Additionally, our algorithm can be seamlessly integrated into clinical information systems, automatically using routine visit data to provide individualized TB risk assessments during subsequent appointments without adding to the physician's workload.

Overall, our findings imply that our score partially assesses intrinsic susceptibility to incident active TB. Hence, it would be compelling to evaluate the score in PWH residing in high TB transmission settings. If our score indeed reflects intrinsic susceptibility, it should effectively identify PWH at the highest risk of TB, even in high-transmission settings. For example, in Peru, BMI serves as a crucial tool for identifying household contacts at elevated risk for active TB, supporting this hypothesis in principle [41].

By employing the Youden cutoff, we established a sensitivity threshold for our ROC curves, enabling direct comparison with established testing methods. Implementing the automated Youden cutoff prevents arbitrary threshold selection that could artificially inflate algorithmic performance. While the Youden cutoff may occasionally yield suboptimal outcomes, prospective clinical testing will allow for refinement and adaptation to specific clinical needs, ensuring optimal performance in real-world settings. In future clinical applications, adjustments to the cutoff can align with specific clinical requirements, emphasizing either high sensitivity or high specificity.

Our study has limitations. The dataset is derived from 2 Central European countries with low MTB transmission rates, potentially introducing selection bias. Moreover, the limited geographical representation may restrict the generalizability of the developed model to more diverse settings. Despite including a total of 98 people with incident active TB and 2435 people without TB, unknown biases may exist that could influence our findings. Incident active TB is typically infrequent, even among PWH [5]. Althouugh we excluded individuals who received preventive therapy, potential biases include the tuberculostatic effects of non-TB drugs such as sulfamethoxazole/trimethoprim prophylaxis, which could impact incident active TB development in PWH [42]. Additionally, people without TB with fluoroquinolone use, which may affect TB development, were not excluded [43].

## CONCLUSIONS

Summarized, the machine learning algorithm introduced in this study has the potential to diagnose PWH at high risk of incident active TB in low transmission settings, surpassing the current clinical standard. Because the algorithm relies on standard clinical assessment during HIV-1 diagnosis and does not require any additional laboratory tests, it holds promise as a highly effective screening method.

## Supplementary Material

ciaf149_Supplementary_Data

## Supplementary Data

Supplementary materials are available at *Clinical Infectious Diseases* online. Consisting of data provided by the authors to benefit the reader, the posted materials are not copyedited and are the sole responsibility of the authors, so questions or comments should be addressed to the corresponding author.

## Notes

***Acknowledgments.*** The data are gathered by 5 Swiss University Hospitals, 2 Cantonal Hospitals, 15 affiliated hospitals, and 36 private physicians (listed in http://www.shcs.ch/180-health-care-providers). The authors thank the patients who participated in the Swiss HIV Cohort Study; the physicians and study nurses for the excellent patient care provided to participants; Jan Meier, Yves Schäfer, and Océane Follonier from the Swiss HIV Cohort Study data center for data management; and Danièle Perraudin and Marianne Amstad for administration.

***Financial support.*** This work was supported by the Swiss National Science Foundation (SNSF) in the framework of the Swiss HIV Cohort Study (grant number 201369), the Swiss HIV Cohort Study (project P 881), and the Swiss HIV Research Foundation.
