# Supplementary material for: Machine Learning-based Prediction of Active Tuberculosis in People With HIV Using Clinical Data
Source: Clin Infect Dis. 2025 Mar 25;81(3):521–30. doi: 10.1093/cid/ciaf149 (PMC12497954; doi:10.1093/cid/ciaf149)
Supplement: ciaf149_Supplementary_Data [file ciaf149_supplementary_data.docx]

**Supplemental Material**

**Supplemental Methods**

**Extended Secondary Outcomes**

Variable importance analysis identified key predictors for incident TB. This was done by using the included Variable Importance ranking in the random forest package in R through the mean decrease in accuracy, which is measured by removing the association between a predictor variable and the outcome variable and determining the resulting increase in error. The effect on the number of TB manifestations prevented as well as the Number Needed to Diagnose provided an estimate of tests needed per diagnosed patient.

**The Random Forest Package**

The randomForest package enables the construction, training, and tuning of a random forest model. This process involves the input of training data and specification of the outcome variable, which, in this case, pertains to the progression to incident active tuberculosis (TB) or its absence. Various hyperparameters can be defined, including *mtry* (the number of features or predictors selected for each tree) and *ntree* (the number of trees constructed for each iteration of the forest). The random forest algorithm extends the classification tree model by generating multiple decision trees and employing bootstrap aggregation (bagging) along with majority voting to reach a decision. This methodology allows the classification of previously unseen data. For instance, in the context of TB prediction, the model provides an estimate of the probability that a participant will develop incident TB in the future. Random forests are based on decision tree models and offer several advantages. These include ease of use and interpretability, robustness to outliers (where statistical fluctuations remain minimal even in the presence of outliers), and the ability to handle missing data. However, a primary limitation of this model is its tendency to overfit, meaning it may perform well on training data but struggle to generalize to new, unseen data. This issue is mitigated through bootstrapping and aggregation: individual trees independently produce decisions, which are then pooled, with the final classification determined by the most frequently selected outcome.

**Model Optimization: Hyperparameters**

We determined that 7 was the optimal number of features for each tree, calculated as the square root of the available predictors (√47 = 6.856, rounded to 7), which is a common method to attain the number of features in each tree. (20) Grid search confirmed this finding, indicating decreased model accuracy with more than 10 features per tree. Additionally, we found 500 to be the optimal number of iterations for trees, as fewer trees resulted in worse and less consistent AUC outcomes, while more iterations provided minimal benefit.

**Sensitivity Analysis**

We conducted a Sensitivity Analysis to assess how the model's Accuracy varies with different latency times to the active outbreak. People with incident active TB were divided into two groups: one experienced an outbreak within 4 years, and the other became active after 4 or more years. This cutoff was determined by analyzing the distribution of time to TB, using the mean of the SHCS to delineate the two groups. This analysis was performed in both the SHCS Model, encompassing all parameters, and the externally Validated cohort. A clear disparity in prediction power was observed: the model validated on outbreaks occurring after 4 or more years performed significantly worse than the one validated with the shorter latency period. (Supplemental Figures 3a+b, 4a+b)

**Imputation (Top Variables)**

A complete people with incident active TB version of the random forest was executed using only the top 20 variables to evaluate the effect of imputation on model performance or variable importance. The imputation had a slight impact on performance, so it was decided to retain it in the bootstrapping (Step one) to reduce the randomness introduced by the Random Forest Imputation.

**Software and Packages Used**

We employed R version 2022.02.3 and RStudio version 2022.02.3+492 "Prairie Trillium" Release for macOS, accessed via the RStudio interface for data management and analysis (22).

**Data sharing statement**

The individual-level data sets generated or analyzed during the current study do not fulfill the requirements for open data access:

1. The SHCS informed consent states that sharing data outside the SHCS network is only permitted for specific studies on HIV infection and its complications, and for researchers who have signed an agreement detailing the use of the data and biological samples; and
2. The data are too dense and comprehensive to preserve patient privacy in persons living with HIV.

According to Swiss law, data cannot be shared if data subjects have not agreed, or data are too sensitive to share. Investigators with a request for selected data should send a proposal to the respective SHCS address ([www.shcs.ch/contact](http://www.shcs.ch/contact%22%20/t%20%22_blank)). The provision of data will be considered by the Scientific Board of the SHCS and the study team.

**Supplemental Results**

**Top 20 predictors**

We selected the top 20 predictors from our model, based on their mean decrease in accuracy.

Subsequently, we selected the same predictors available in the external validation cohort (AHIVCOS: Austrian HIV Cohort Study), except for profession, which is not recorded in the validation cohort, and CD3 and CD8 percentage, due to excessive missing data during the 6 months around registration. This yields a very similar result to the model containing all parameters, making it feasible to only use the reduced set. This also facilitated choosing parameters in the validation cohort.

| **Supplemental Table 1: Included variables**  List of all 48 variables included in the first model (full model with SHCS data). Abbreviations: HIV, Human Immunodeficiency Virus, ID, Identification | |
| --- | --- |
| **Variable** | **Description** |
| Hemoglobin | Grams per deciliter |
| Platelets | 10^9^ /liter |
| Leucocytes | Cells/microliter |
| CD3-Count | Cells/microliter |
| CD4-Count | Cells/microliter |
| CD8-Count | Cells/microliter |
| CD8-Percentage | CD8 as % of lymphocytes |
| Ability to Work | In 0-100% Employment |
| Mean Arterial Pressure (MAP) | mmHg |
| Cholesterol | mmol/liter |
| High-density lipids (HDL) | mmol/liter |
| Triglycerides | mmol/liter |
| Glucose (in blood) | mmol/liter |
| Creatinine | µmol/liter |
| Body-Mass-Index (BMI) | kg/m^2^ |
| Toxoplasmosis test result | Positive or negative or unknown |
| Ag-HBs-test result | Positive or negative or unknown |
| Type of virus detected | HIV-1, HIV-2, other |
| RNA | Log10 of copies per milliliter |
| Hypertension in medical history | Yes/No/unknown |
| Patient currently smokes | Yes/No/unknown |
| Smoking in medical history | Yes/No/unknown |
| Pretreated for HIV | Yes/No/unknown |
| Patient lives alone | Yes/No/unknown |
| Hospitalization in the last 6 months | Yes/No/unknown |
| Results of the Anti-HBs-Test | Positive or negative or unknown |
| Fat loss (in the face, arms, legs, buttocks, abdomen, breasts, or neck) | Yes/No/unknown |
| Fat accumulation (in the face, arms, legs, buttocks, abdomen, breasts, or neck) | Yes/No/unknown |
| Positive family history of heart ailments | Yes/No/unknown |
| Previous HIV test | Yes/No/unknown |
| Previous negative HIV result | Yes/No/unknown |
| Stable partner | Yes/No/unknown |
| Occasional partners | Yes/No/unknown |
| Result of the Anti-HBc-test | Positive or negative or unknown |
| Risk group for most likely exposure to HIV | 1-9 e.g., 1 = homosexual contacts, 4 = i.v. drug use (with needle sharing) |
| Sexual preference | Homosexual, bisexual, heterosexual, unknown |
| Education (highest completed level of education) | 1-9 e.g., 1= no completed school, 7 = higher education |
| Profession (last held position) | 1-9 e.g., 1= self-employed, 7 = houseman/-wife |
| First Source (source of information) | 1-4 e.g., 1 = from this cohort center, 3= private physician |
| First Center | Center where they first joined the SHCS |
| Setting (category of visit) | 1-3 e.g., 1= outpatient visit, 3 = no visit |
| Region of Origin | WHO-Code e.g., 005 = South America |
| Ethnicity | 0-9, e.g., 1= white, 4 = Asian |
| Cytomegalovirus | Positive or negative or unknown |
| Sex | Female/Male |
| Intravenous drug-use | Yes/No/unknown |
| Age at registration | In years |
| Syphilis test result | Positive or negative or unknown |

| **Supplemental Table 2: Excluded Variables**  List of all variables excluded due to not meeting the criteria of having more then 65% of the data points available during the chosen time frame of ± 6 months around date of registration | |
| --- | --- |
| Lactate | mmol/l |
| Alanine-Aminotransferase | IU/l |
| Alk. Phosphatase | IU/l |
| Quick | INR |
| Bilirubin | µmol/l |
| Albumin | g/l |
| Pancreatic amylase | IU/l |
| Aspartate-Aminotransferase | IU/l |
| Glucose in Urine, Strip test | Pos/neg |
| Protein in Urine, Strip test | Pos/neg |
| Glucose in Urine, meth Standard | mg/dl |
| Protein in Urine, meth Standard | g/l |
| Amylase nonspecific | IU/l |
| Creatinine in urine | mmol/l |
| Protein/Creatinine in urine | - |
| Creatinine Kinase in serum | U/l |
| Gamma glutamyl transferase | U/l |
| Cystatin C | mg/l |
| Glycated hemoglobin HBA1c | (%) |
| Tuberculin Skin Test | Pos/neg/uncertain |
| Interferon gamma release assay | Pos/neg/uncertain |
| Hepatitis D Testing | - |
| Hepatitis E Testing | - |
| Is the patient aware of the source of his HIV infection | Yes/no/doesn’t wish to answer |
| Can the patient give information about the time of his HIV infection? | Yes/no/doesn’t wish to answer |
| Where did the infection most likely occur? | Switzerland, while temporarily abroad, as a resident abroad, unknown |
| Information on earnings of the patient |  |
| Number of hours the patient really worked during the last month | 42h/week = 100% |
| Was a screening for sexually transmitted infections done? | Yes/no/unknown |
| Did the patient have a clinical event/DEXA/fibroscan/biopsy/resistance test/ serious non opportunistic infection/STD/Anal Cancer Screening since the last Follow-up? | Yes/no/unknown  Every point separated by a slash (/) is encoded as a separate variable |
| Did the Patient consume cannabis/cocaine/heroine? | Yes/no/ doesn’t wish to answer  Every point separated by a slash (/) is encoded as a separate variable |
| Does the patient suffer from depression? | Yes/no/unknown |
| Was the patient imprisoned since the last visit? | Yes/no/ doesn’t wish to answer |
| Is the patient in a drug substitution program? | Yes/no/ doesn’t wish to answer |
| Did the patient travel to the tropics? | Yes/no/ doesn’t wish to answer |

**Supplemental Figure 1**

**
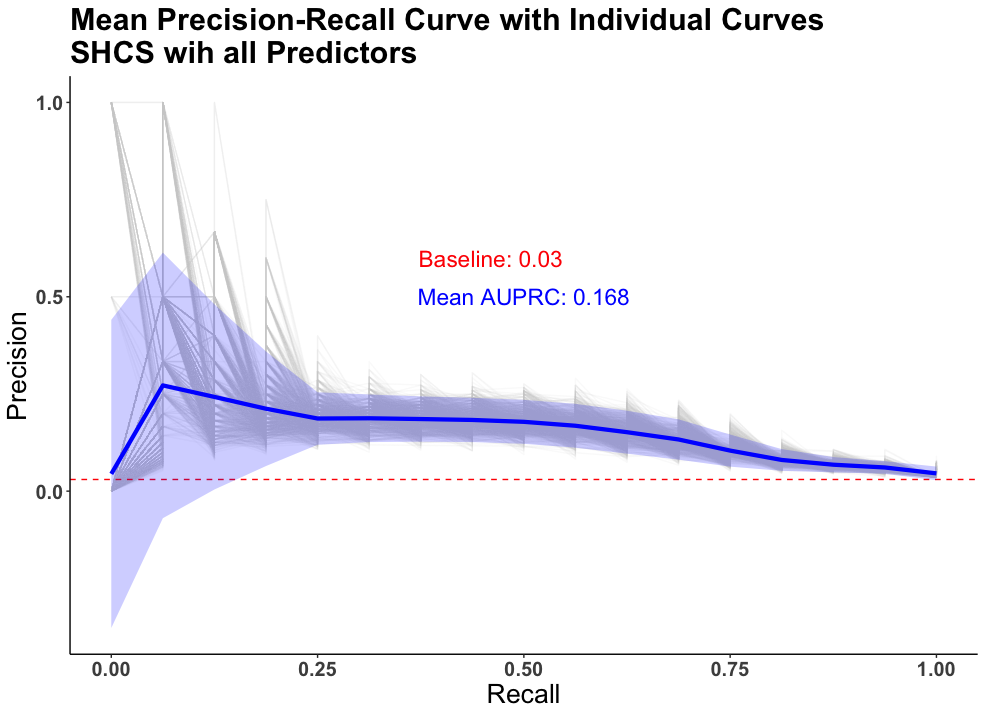
**

**Supplemental Figure 1**: Mean area under the precision recall curve with standard deviation margins of the complete SHCS model that includes all parameters. With a baseline of 0.03 that is the prevalence of people with incident active TB in the model population (1:31). This curve looks at how well the model can identify true positives, completely ignoring the true negative rate to give a better understanding of the precision of identifying real instances of people with incident active TB in an unbalanced data set. *Abbreviations: SHCS: Swiss HIV Cohort Study, HIV: Human immunodeficiency virus, AUPRC: Area under the Precision Recall Curve*

***ALT TEXT Supplemental Figure 1:*** *The mean area under the precision recall curve with standard deviation margins of the complete Swiss HIV Cohort Study model that includes all parameters is presented. The curve is above the baseline of 0.03, with a mean of 0.168, hence showing the predictive performance of the model.*

**Supplemental Figure 2**

**
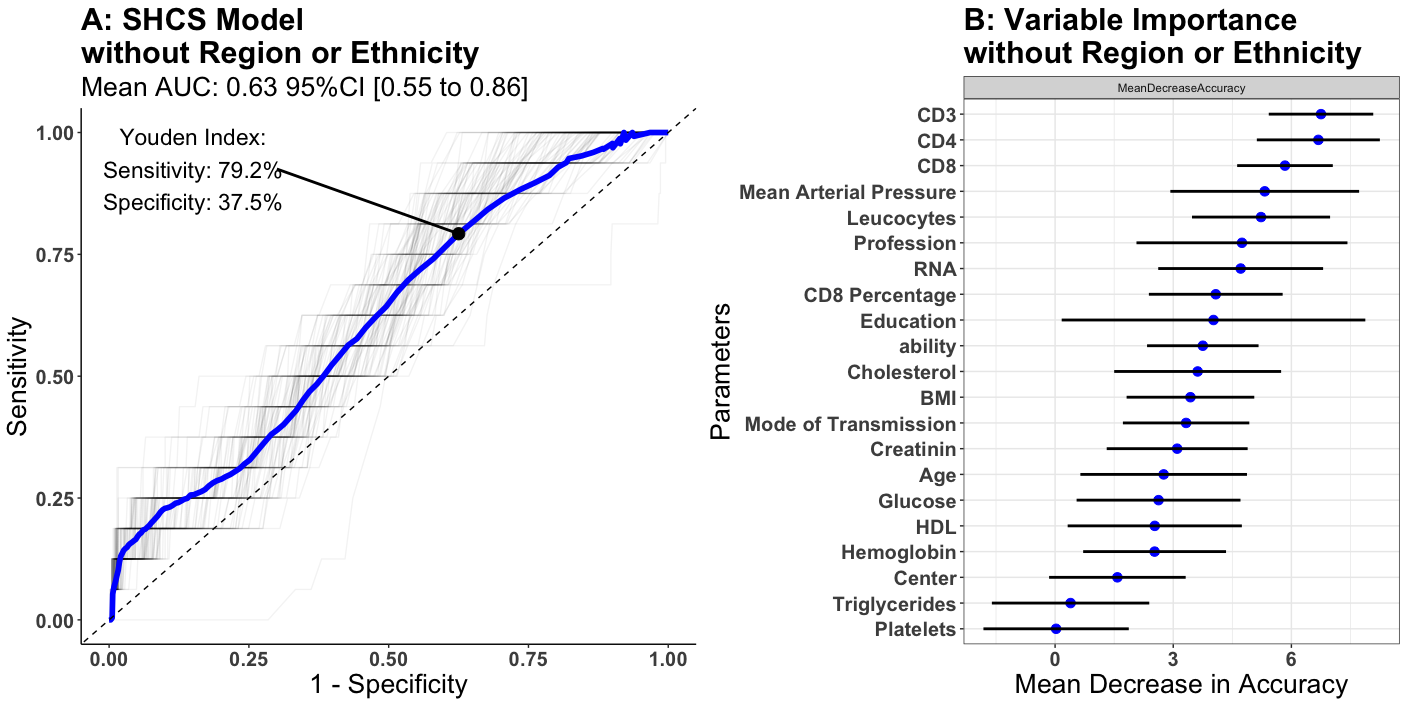
**

**Supplemental Figure 2a**: Receiver operating characteristic (ROC) curve for the SHCS chosen variables excluding ‘Ethnicity’ and ‘Region of Origin’ of the random forest model. The Youden Index, calculated as (Sensitivity + Specificity - 1), was used to determine the optimal point on the ROC curve.

**Supplemental Figure 2b**: Variable importance of the model, with ‘Ethnicity’ and ‘Region of Origin’ excluded as parameters. *Abbreviations: HIV: Human immunodeficiency virus, SHCS: Swiss HIV Cohort Study, AUC: Area under the Curve, BMI: Body Mass Index, HDL: High-Density Lipoproteins, RNA: Ribonucleic acid, CD (): Cluster of Differentiation*

***ALT TEXT Supplemental Figure 2:*** *Split into two parts, the model results without including information on ethnicity or region of origin are shown. On the left it is seen that the area under the receiver operating characteristics curve is 0.63, with the Youden Index indicating a sensitivity of 79.2% and a specificity of 37.5%. On the right, the variables are listed according to the importance in the model, with CD3, CD4, and CD8 on top.*

**Supplemental Figure 3**


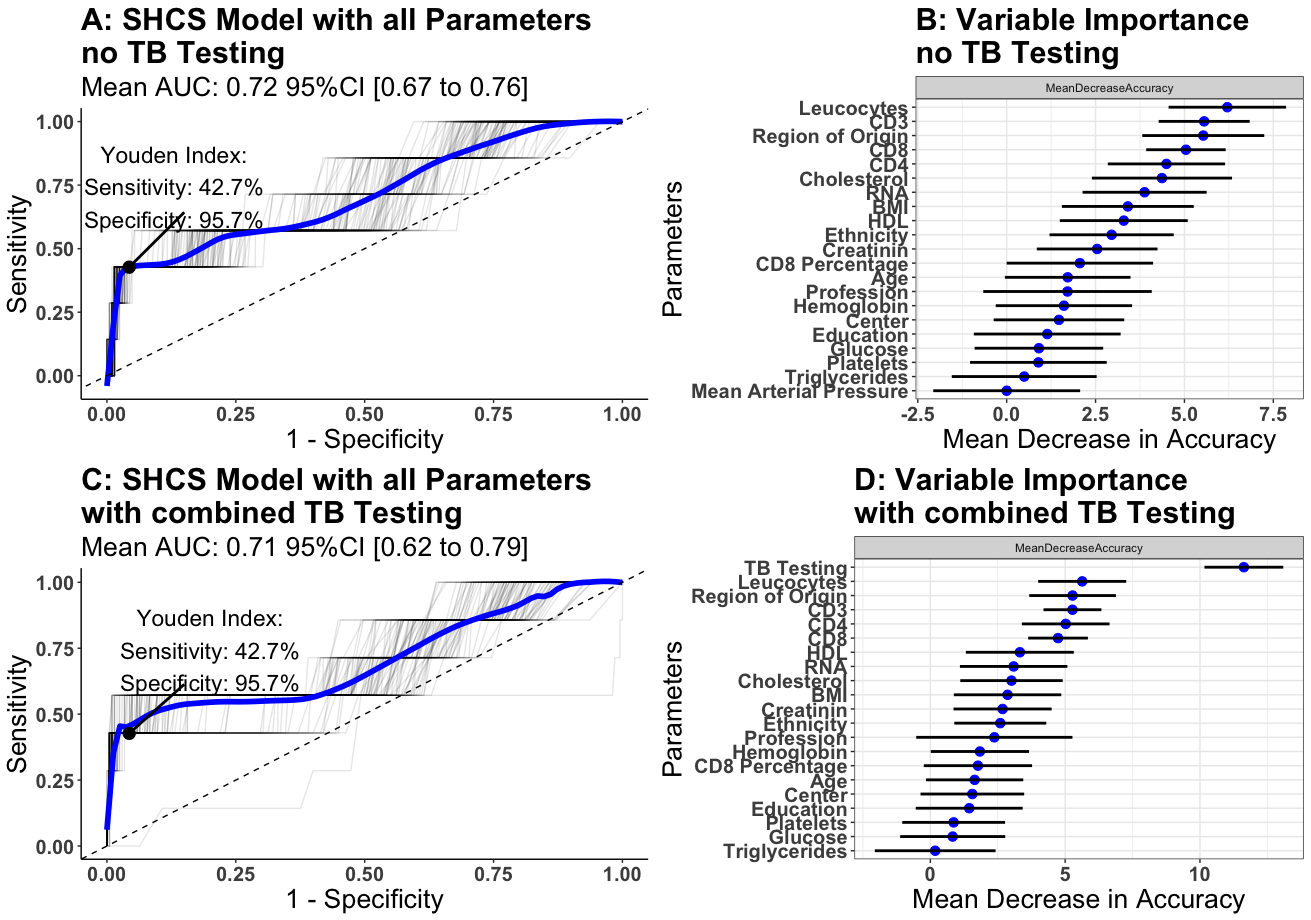
 **Supplemental Figure 3a**: Receiver Operating Characteristic (ROC) curve for the SHCS model restricted to people with IGRA or TST testing, but excluding their result, of the Random Forest model. The Youden Index, calculated as (Sensitivity + Specificity - 1), was used to determine the optimal point on the ROC curve.

**Supplemental Figure 3b**: Variable importance of the model predicting incident active TB in the subset of people with available IGRA or TST result but excluding the actual result.

**Supplemental Figure 3c**: Receiver Operating Characteristic (ROC) curve for the SHCS chosen variables with the People with incident active TB with IGRA or TST testing, including their result, of the Random Forest model The Youden Index, calculated as (Sensitivity + Specificity - 1), was used to determine the optimal point on the ROC curve.

**Supplemental Figure 3d**: Variable importance of the model predicting incident active TB in the subset of people with available IGRA or TST result, including the actual result as parameter.

*Abbreviations: HIV: Human immunodeficiency virus, SHCS: Swiss HIV Cohort Study, AUC: Area under the Curve, BMI: Body Mass Index, HDL: High-Density Lipoproteins, RNA: Ribonucleic acid, CD (): Cluster of Differentiation*

***ALT TEXT Supplemental Figure 3:*** *Split into four parts, the model results with and without including information on tuberculosis testing are presented. On the left, the area under the receiver operating characteristics curve is presented, first without including information on tuberculosis testing, then with this information included. It is shown that the curves are almost identical. On the right, the variable importance information of the respective models is shown, first without information on tuberculosis testing, then with this information included. Interestingly, although the area under the curves is similar, the variable importance is different between the two models, with information on tuberculosis testing even being the most important variable in the latter model.*

**Supplemental Figure 4**


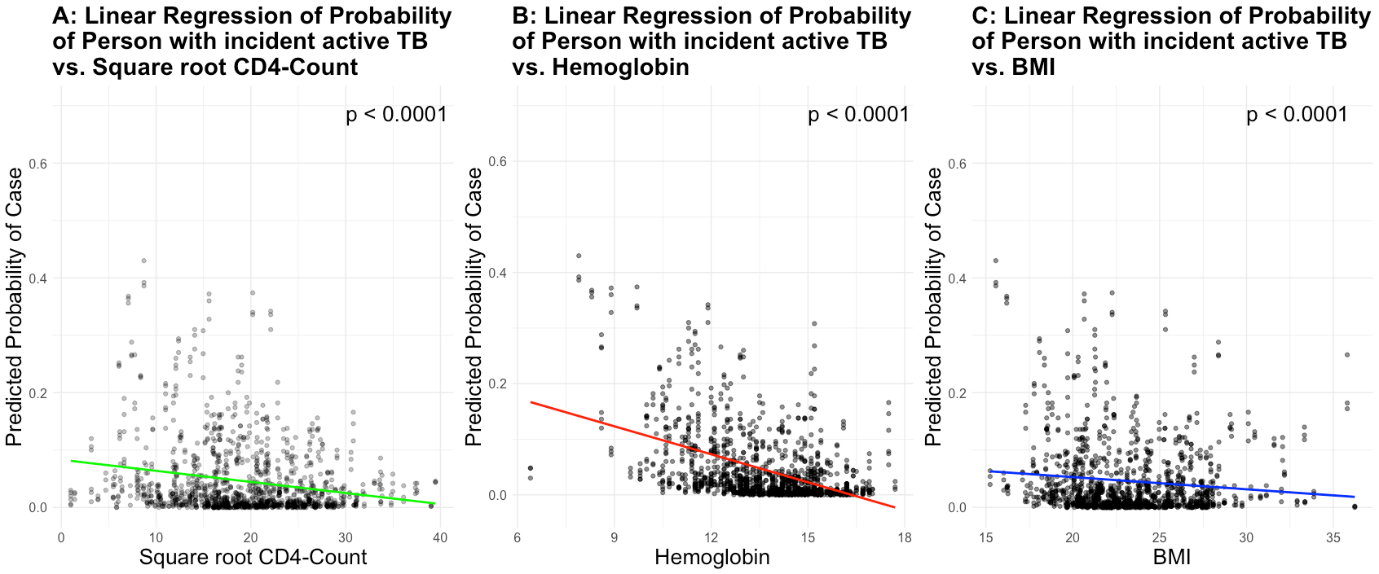


**Supplemental Figure 4a**: This Figure shows a scatterplot that illustrates the relationship between the square root of the CD4 count and the predicted probability of being classified as a person with incident active TB. The green line represents the fitted linear regression and demonstrates a statistically significant (p < 0.0001) correlation between the two, suggesting a fall in CD4 count increases the likelihood of it being a person with incident active TB.

**Supplemental Figure 4b**: This Figure shows a scatterplot that illustrates the relationship between Hemoglobin (g/dl) and the predicted probability of being classified as a person with incident active TB. The red line represents the fitted linear regression and demonstrates a statistically significant (p < 0.0001) correlation between the two, suggesting a fall in Hemoglobin increases the likelihood of it being a person with incident active TB.

**Supplemental Figure 4c**: This Figure shows a scatterplot that illustrates the relationship between BMI (kg/m2) and the predicted probability of being classified as a person with incident active TB. The blue line represents the fitted linear regression and demonstrates a statistically significant (p < 0.0001) correlation between the two, suggesting a fall in BMI increases the likelihood of it being a person with incident active TB.

*Abbreviations: BMI: Body Mass Index, CD (): Cluster of Differentiation*

***ALT TEXT Supplemental Figure 4:*** *Split into three parts, the linear correlations of the precited probability for developing active tuberculosis and CD4 cell count, hemoglobin, and body mass index, respectively, are shown. For all three parameters, there is a significant linear trend, with lower CD4 cell count, lower hemoglobin and lower body mass index being correlated with a higher probability for developing active tuberculosis.*

**
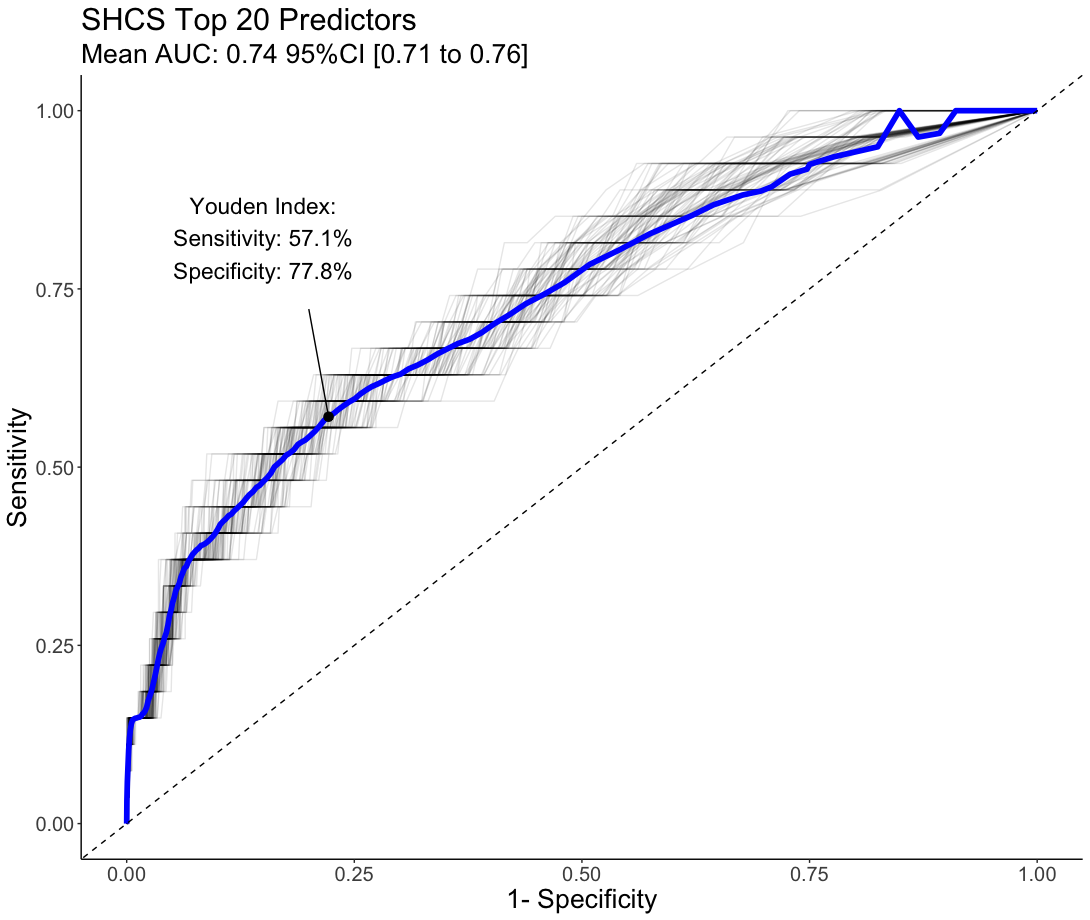
Supplemental Figure 5**

**Supplemental Figure 5**: Receiver operating characteristic (ROC) curve for the incident active tuberculosis of the random forest model, built using the top 20 parameters from the SHCS data. The Youden Index, calculated as (Sensitivity + Specificity - 1), was used to determine the optimal point on the ROC curve.

*Abbreviations: HIV: Human immunodeficiency virus, SHCS: Swiss HIV Cohort Study, AUC: Area under the Curve*

***ALT TEXT Supplemental Figure 5:*** *The receiver operating characteristic curve for the random forest model built using the top 20 parameters from the SHCS data is presented. The area under the curve is 0.74, and the Youden Index gives a sensitivity of 57.1% and a specificity of 77.8%.*

**Supplemental Figure 6**


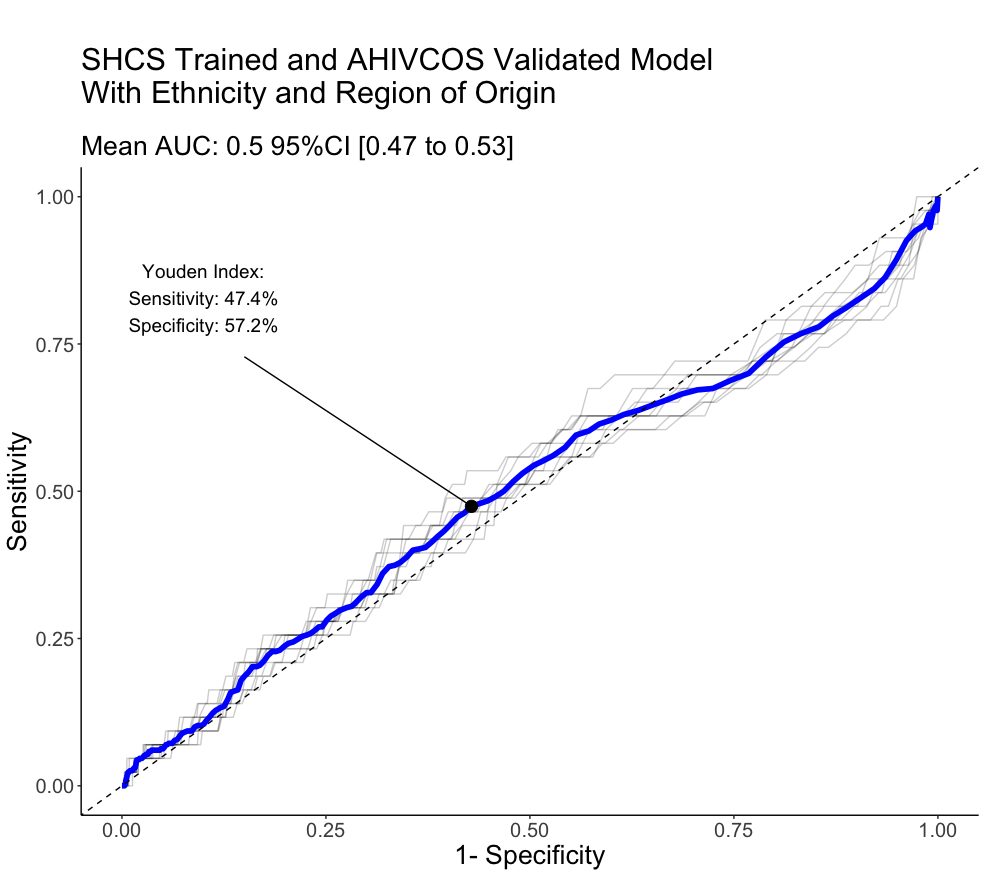


**Supplemental Figure 6**: The receiver operating characteristic curve (ROC) for predicting incident active TB using the random forest model with the top 20 parameters, including ‘Ethnicity’ and ‘Region of Origin’ from the SHCS data, in the validation cohort, i.e., AHIVCOS. The Youden Index, calculated as (Sensitivity + Specificity - 1), was used to determine the optimal point on the ROC curve.

*Abbreviations: SHCS: Swiss HIV Cohort Study, AHIVCOS: Austrian HIV Cohort Study, HIV: Human Immunodeficiency Virus, AUC: Area under the Curve*

***ALT TEXT Supplemental Figure 6:*** *The receiver operating characteristic curve for the original random forest model as shown in the main manuscript is presented, validated with data from the Austrian HIV Cohort Study. The area under the curve is 0.5, and the Youden Index gives a sensitivity of 47.4% and a specificity of 57.2%.*
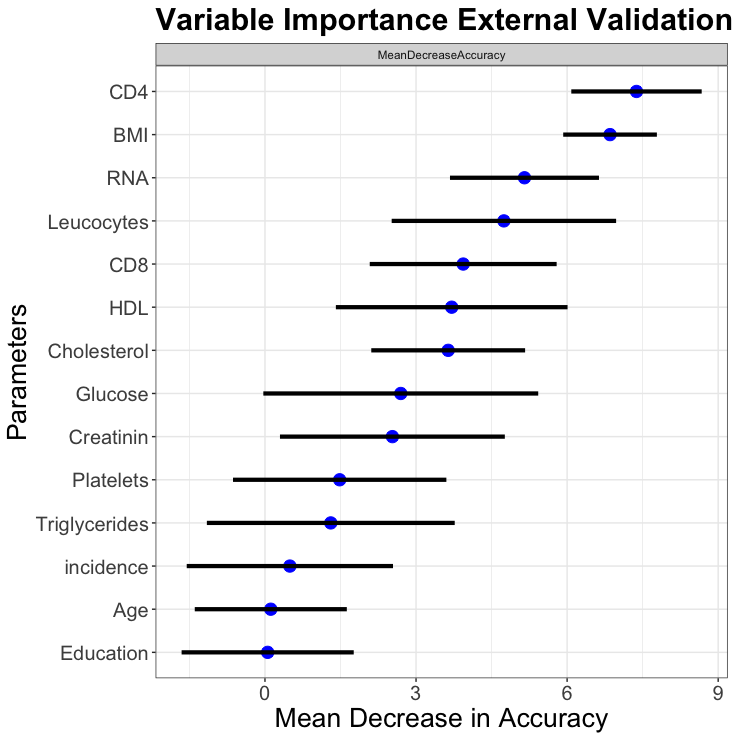
**Supplemental Figure 7**

**Supplemental Figure 7**: Variable importance of the external validation model, with ‘Ethnicity’ and ‘Region of Origin’ excluded as parameters. The variables are sorted by Mean Decrease Accuracy, which is the measure of the decrease in accuracy when the association between a predictor variable and the outcome variable is removed. This measure determines the resulting increase in error. *Abbreviations: BMI: Body Mass Index, HDL: High-Density Lipoproteins, RNA: Ribonucleic acid, CD (): Cluster of Differentiation*

***ALT TEXT Supplemental Figure 7****: The variable importance of the external validation model, with ‘Ethnicity’ and ‘Region of Origin’ excluded as parameters, is presented. On top is CD4 cell count, followed by body mass index.*

**Supplemental Figure 8a+b**

**Supplemental Figure 8A**: Mean area under the precision recall curve with standard deviation margins of the SHCS model validated with SHCS data with the dataset limited to the top 20 predictors. With a baseline of 0.03 that is the prevalence of people with incident active TB in the study population (1:31).

**Supplemental Figure 8B**: Mean area under the precision recall curve with standard deviation margins of the SHCS model validated with AHIVCOS data with the dataset limited to the top 20 predictors. With a baseline of 0.03 that is the prevalence of people with incident active TB in the study population (1:31).

*Abbreviations: SHCS: Swiss HIV Cohort Study, HIV: Human immunodeficiency virus, AUPRC: Area under the Precision Recall Curve, AHIVCOS: Austrian HIV Cohort Study*

***ALT TEXT Supplemental Figure 8:***  *Split into two parts, the mean area under the precision recall curve with standard deviation margins for the Swiss HIV Cohort Study model with the top 20 variables are presented. To the left for the validation with the Swiss HIV Cohort Study data (value is 0.11), to the right for the Austrian HIV Cohort Study (value is 0.095).*

**
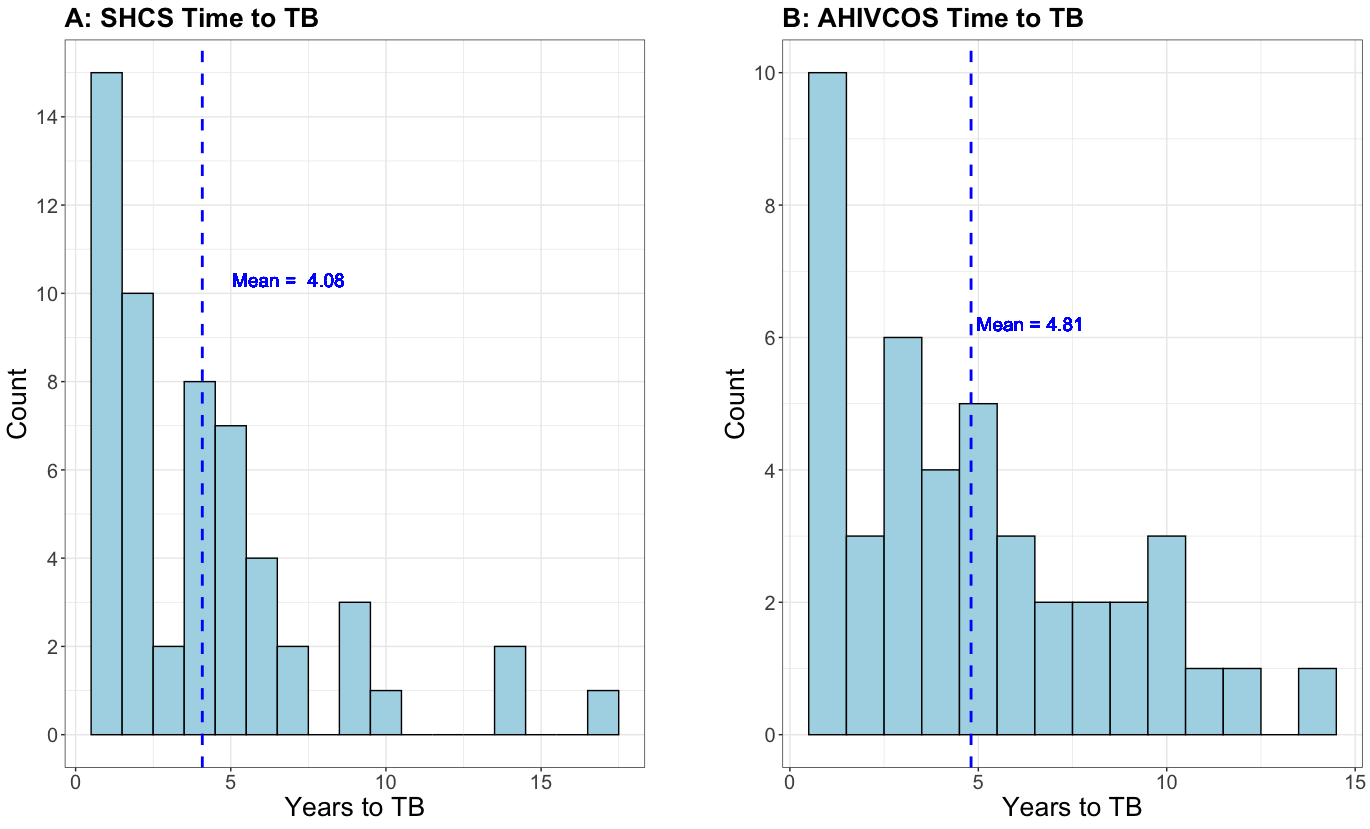
Supplemental Figure 9a+b**

**Supplemental Figure 9a**: Years to an outbreak of incident TB from the year of registration in PWH enrolled in the SHCS.

**Supplemental Figure 9b**: Years to the outbreak of incident TB from the year of registration in PWH enrolled in the AHIVCOS.

*Abbreviations: SHCS: Swiss HIV Cohort Study, HIV: Human Immunodeficiency Virus, TB: tuberculosis, incident TB: the outbreak of TB ≥ 6 months after registration, PWH: People with HIV, AHIVCOS: Austrian HIV Cohort Study*

***ALT TEXT Supplemental Figure 9:*** *Split into two parts, the distribution of the number of years before active tuberculosis is shown in the form of a bar plot. On the left, numbers for the Swiss HIV Cohort Study (mean = 4.08 years) are shown, the right for the Austrian HIV Cohort Study (mean = 4.81 years).*
